# Effect of Health Education on Knowledge and Attitudes Toward Reproductive Health Among Pre-university Girls in an Urban Area

**DOI:** 10.7759/cureus.76354

**Published:** 2024-12-25

**Authors:** Vaishali R N, Girija J Mahantshetti, Sulakshana S Baliga, Anjali J Patil

**Affiliations:** 1 Community Medicine, Arunai Medical College and Hospital, Tiruvannamalai, IND; 2 Community Medicine, Jawaharlal Nehru Medical College, Belagavi, IND

**Keywords:** adolescent health, health intervention, menstruation, puberty, reproductive literacy

## Abstract

Background: For every woman, menstruation is a natural physiological process, and the adolescence period marks the beginning of the menstruation process. Investing the right knowledge in girls at a young age directs a better future for women, children, and families, thereby leading to intergenerational impact.

Objective: This study aims to evaluate the impact of health education on reproductive health among pre-university girls in an urban area.

Methodology: An interventional study was conducted among 500 girls studying in pre-university colleges (PUC). A pre-test was given to assess the baseline knowledge and attitude regarding reproductive health using a questionnaire followed by an interactive health education session. After one month, the impact of health education was assessed by administering the post-test questionnaire. The statistical software IBM SPSS Statistics for Windows, Version 20 (Released 2011; IBM Corp., Armonk, New York, United States) was used for the analyses.

Results: This study revealed that after the educational intervention, there was a significant improvement from the pre-test to the post-test. The median knowledge score increased by 6 points with a Z value of -16.93 and r = 0.53, and the median attitude score improved by 4 points with a Z value of -9.09 and r = 0.287. These results were found to be statistically significant.

Conclusion: Educational interventions for the targeted group significantly improved the knowledge and attitude regarding reproductive health among PUC girls.

## Introduction

For every woman, menstruation is a natural physiological process, and adolescence marks the beginning of the menstruation process, where females have to prepare and adjust themselves to manage them [1]. As per the World Health Organization, those who are 10-19 years old belong to the adolescent category, and this phase brings out both biological and psychological growth in children [2]. Globally, this stage is critical, as puberty, which occurs during adolescence, marks a time of increased susceptibility to leaving school, early marriage, and teenage pregnancy [3]. India has nearly 250 million adolescents, of which 45% of them are females, according to the 2011 census [4]. According to the National Family Health Survey 5, only 77.3% of women aged 15-24 years use hygienic methods of protection during their menstrual period [5].

In this society, menstrual hygiene is neither properly acknowledged nor received adequate attention. This inadequate knowledge leads to negative implications for female health and requires special attention because of the physical and emotional problems associated with it [6]. Several belief and cultural taboos have been the problem for both adolescent girls and women in both rural and urban areas. Also, discussing sexual and reproductive health within and between generations is restricted by several cultural taboos [7].

Other major problems in the adolescent age group are child marriage, early pregnancy, and childbirth. Approximately 16% of 15-19-year-old girls are presently married [8]. In India, there is very little awareness among adolescent girls regarding reproductive health, as there is social prohibition and negative attitudes among parents in discussing these matters with them, thereby blocking access to the right information [9]. Health education given to adolescent girls improves their knowledge and encourages them to improve and maintain their health, prevent diseases, and reduce risky behaviors [10]. It is important that despite government efforts to improve reproductive health, many adolescents were unaware of crucial topics like menstruation, antenatal care, and contraception. Investing in the right knowledge in girls at a young age directs a better future for women, children, and families, thereby leading to intergenerational impact [11]. This study was therefore conducted to evaluate the impact of a health education intervention on pre-university girls' knowledge and attitudes about reproductive health in an urban setting. After the health education intervention, the knowledge and attitude of pre-university girls were assessed again to see the impact.

## Materials and methods

Study design, duration, and sample size calculation

The study was an interventional study conducted from March 2021 to May 2022. Using G Power software (Heinrich-Heine-Universität Düsseldorf, Düsseldorf, Germany) for the statistical test by the difference between two dependent means (matched pairs), the sample size was calculated by assuming the effect size to be 0.25. The false positive (α) was set at 0.01, with a power of the test (1-β) of 0.99 and β = 0.01. Based on these parameters, the sample size (n) was calculated to be 388. Considering attrition, with a factor of 1.2 for lost follow-up, the adjusted sample size was n = 388 x 1.2, resulting in n = 465, which was rounded up to 500. Therefore, the total sample size was 500.

Study population

The list of all the pre-university colleges (PUC) in the city's urban area was obtained from the Deputy Director of Public Instructions (DDPI) office. There were 46 PUCs in urban areas. Girls studying in I and II PUCs were randomly selected from four colleges (125 girls from each college), one from each zone (North, South, East, West) for the study. Adolescent girls who had attained menarche and those who gave consent were enrolled in the study.

Sampling method

The sampling frame was made, and each participant was selected by the systematic random sampling method with a sampling interval (k) of 3. A total of 125 girls from each PUC were selected: 62 girls from PUC I and 63 girls from PUC II.

Permission from the principal of each PUC was obtained, and written informed consent was obtained from parents, along with assent from the girls.

Study tool

Data was collected regarding socio-demographic variables, knowledge and attitude about puberty and menstruation, antenatal care, and contraception. A paper-based pre-test questionnaire was administered to assess students’ knowledge and attitudes on reproductive health. To ensure overall reliability, the questionnaire was internally validated using Cronbach's alpha, and the cumulative value obtained was 0.839, which indicates good internal consistency. Knowledge was assessed by multiple-choice questions (correct responses received a score of one, while incorrect responses received zero), and attitude was assessed using a 5-point Likert scale (strongly disagree, disagree, neutral, agree, and strongly agree).

A pre-test was followed by a health education session. Health education was given using audio-visual aids such as lectures, PowerPoint presentations, videos, and flip charts for one hour, followed by asking and answering questions in an interactive session where the students’ queries were clarified. The content for the health education session was validated by the Department of Community Medicine. After one month, the impact of health education on knowledge and attitudes toward reproductive health was assessed by administering the post-test questionnaire.

Ethical considerations

Ethical clearance was obtained from the Institutional Ethical Committee, Jawaharlal Nehru Medical College, KLE Academy of Higher Education and Research (KAHER) (reference number: MDC/JNMCIEC/73).

Statistical analysis

The statistical analysis was done using IBM SPSS Statistics for Windows, Version 20 (Released 2011; IBM Corp., Armonk, New York, United States). For categorical variables, percentages and frequencies were used. To test the normality of the data, the Kolmogorov-Smirnov test was used. As the data was not normally distributed, the Wilcoxon signed-rank test was used to determine the difference between pre-test and post-test results with p-value < 0.05 considered as statistically significant.

## Results

The participants' mean age was 17.15 ± 0.7 years. The majority (89.2%) identify as Hindu. Most (72.6%) came from nuclear families, and 90.6% lived with both parents. In terms of living conditions, 60.8% resided in urban areas, 89.2% lived in pucca houses, and 81.8% had indoor sanitary privies. Additionally, 55.8% belonged to the class III socioeconomic scale (SES) according to the modified B.G. Prasad classification. About 66% of the study participants attained their menarche between the ages of 10 and 15 years, and the mean age of menarche among the study participants was 11.4 ± 5.2 years. During their first menstruation, 61.4% experienced abdominal and back pain, 19.2% reported sleeplessness, and 18.8% noted heavy bleeding (Table 1).

**Table 1:** Socio-demographic characteristics of study participants (n = 500) PUC: pre-university college

Socio-demographic variables		Frequency (%) n = 500 (%)
Age of the student (in years)	<16	1 (0.2%)
	16-18	493 (98.6%)
	>18	6 (1.2%)
Class	PUC I	264 (52.8%)
	PUC II	236 (47.2%)
Educational qualification of mother	Illiterate	17 (3.4%)
	Completed 8th standard	85 (17%)
	10th-12th standard/diploma	280 (56%)
	Graduates/post graduates	118 (23.6%)
Occupation of mother	Home maker	404 (80.8%)
	Government employee	26 (5.2%)
	Private employee	23 (4.6%)
	Self-employed	47 (9.4%)
Source of information regarding menstruation	Mother	352 (70.4%)
	Teacher	14 (2.80%)
	Friends	42 (8.40%)
	Others	92 (18.4%)

In the present study, during the pre-test, 83.2% of girls said that there should be a session in school regarding information on menstruation, which improved to 93% after the health education intervention session. In the pre-test, only 15% of students were comfortable talking about menstruation-related queries to any teacher, 48.4% were comfortable with teachers of the same gender, and 36.6% were uncomfortable asking menstruation-related queries to any teacher. Following the health education intervention, 24.4% of students were comfortable talking about menstruation-related queries to any teacher, 58.2% were comfortable with teachers of the same gender, and 17.4% were uncomfortable asking menstruation-related queries to any teacher (Table 2).

**Table 2:** Knowledge regarding menstruation among the study participants (n = 500) Data shown are frequencies (n) of subjects and proportions (%). *McNemar test, p < 0.05, indicates statistical significance.

	Items	Pre-test	Post-test	p-value
1.	Menstruation is a physiological process	248 (49.6%)	405 (81%)	<0.001*
2.	Blood comes out through vagina during menstruation	316 (63.2%)	432 (86.4%)	0.001*
3.	There should be session in school regarding information on menstruation	416 (83.2%)	465 (93%)	<0.001*
4.	Normal duration of menstrual blood flow (2-7 days)	441 (88.2%)	468 (93.6%)	0.005*
5.	Normal interval between two menstrual cycles (21-35 days)	348 (69.6%)	426 (85.2%)	<0.001*
6.	Commercially made sanitary pad should be used during menstruation	399 (79.8%)	470 (94%)	<0.001*
7.	Frequency of changing absorbent (4-6 hours once)	201 (40.2%)	303 (60.6%)	<0.001*
8.	Dustbin should be used for disposal of sanitary pads	446 (89.2%)	477 (95.4%)	<0.001*
9.	Paper should be used as wrap during disposal of sanitary pads	321 (64.2%)	406 (81.2%)	<0.001*
10.	Cleaning of genitalia at every visit to toilet during menstruation	456 (91.2%)	489 (97.8%)	<0.001*

During the pre-test, 30.2% of girls answered that antenatal care helps in promoting healthy lifestyles and preventing probable health issues, which increased to 66.40% after health education intervention. Initially, 60.6% of girls said that it is essential to know about contraception. After the health education session, the proportion increased to 78.4% (Table 3).

**Table 3:** Knowledge regarding pregnancy, antenatal care, and contraception among the study participants (n = 500) Data shown are frequencies (n) of subjects and proportions (%). *p < 0.05 indicates statistical significance

	Items	Pre-test	Post-test	p-value
1.	Recommended age of marriage for girls is 18 years	195 (39%)	332 (66.4%)	<0.001*
2.	First sign of pregnancy is missed period	421 (84.2%)	434 (86.8%)	0.283
3.	Regular medical and nursing care is recommended for women during pregnancy	147 (29.4%)	352 (70.4%)	<0.001*
4.	Proper diet, rest, and mild exercise are essential during pregnancy	461 (92.2%)	472 (94.41%)	0.193
5.	Consuming nutritious foods is beneficial for the health of pregnant women and their babies	480 (96%)	497 (99.4%)	<0.001*
6.	Contraception is the responsibility of both husband and wife	413 (82.6%)	466 (93.4%)	<0.001*
7.	Contraception helps to avoid unwanted pregnancy	287 (57.4%)	429 (85.8%)	<0.001*
8.	Contraception helps to maintain regular interval between pregnancy	36 (7.2%)	228 (45.6%)	<0.001*

After the health education session, the girls demonstrated a positive shift in their attitudes toward reproductive health. During the pre-test, only 41.4% strongly agreed that social and cultural misconceptions and taboos surrounding menstruation, such as restrictions on activities during periods, should be eliminated, which improved to 66% after the health education session (Tables 4-5).

**Table 4:** Attitude regarding menstruation among the study participants (strongly agreed) Data shown are frequencies (n) of subjects and proportions (%).

	Items	Pre-test	Post-test
1.	Parents/teachers should be consulted on the matters of reproductive health education	168 (33.6%)	274 (54.8%)
2.	Every girl needs to learn about menstrual hygiene	347 (69.4%)	371 (74.20%)
3.	Poor menstrual hygiene predisposes to infection	149 (29.8%)	214 (42.8%)
4.	Menstrual problems interfere with academic performance	54 (10.8%)	131 (26.2%)
5.	Cultural and social taboos (not entering kitchen, do not sit in same place as others in house) during menstrual period should be eliminated	207 (41.4%)	330 (66%)

**Table 5:** Attitude regarding pregnancy and antenatal care among the study participants (strongly agreed) Data shown are frequencies (n) of subjects and proportions (%).

	Items	Pre-test	Post-test
1.	Marriage within close relatives should be avoided	175 (35%)	191 (38.2%)
2.	Regular antenatal checkup and follow up is essential during pregnancy	229 (45.8%)	272 (54.4%)
3.	Pregnant women should change dietary habit as advised by doctor	196 (39.2%)	255 (51%)
4.	Using medication without prescription of doctors during pregnancy can be harmful	267 (53.4%)	315 (63%)
5.	Smoking during pregnancy is harmful to baby	365 (73%)	411 (82.2%)

In this study, the Wilcoxon signed rank test shows that there was an improvement from the pre-test to the post-test after the educational intervention, with knowledge scores significantly higher, with a median of differences in scores being +6 and r = 0.53, and attitude scores with a median of differences being +4 and r = 0.287. This was found to be statistically significant (Table 6).

**Table 6:** Comparison of knowledge and attitude scores among the study participants (n = 500) *p < 0.05 indicates statistical significance

Scores	Pre-test		Post-test		Z-score	p-value
	mean ± SD (minimum-maximum)	Median	mean ± SD (minimum-maximum)	Median		
Knowledge score	15.38 ± 3.827 (3-23)	16	20.97 ± 2.89 (11-26)	21	-16.93	<0.001*
Attitude score	38.62 ± 8.50 (0-50)	40	42.74 ± 5.89 (11-50)	44	-9.09	<0.001*

**Figure 1:**
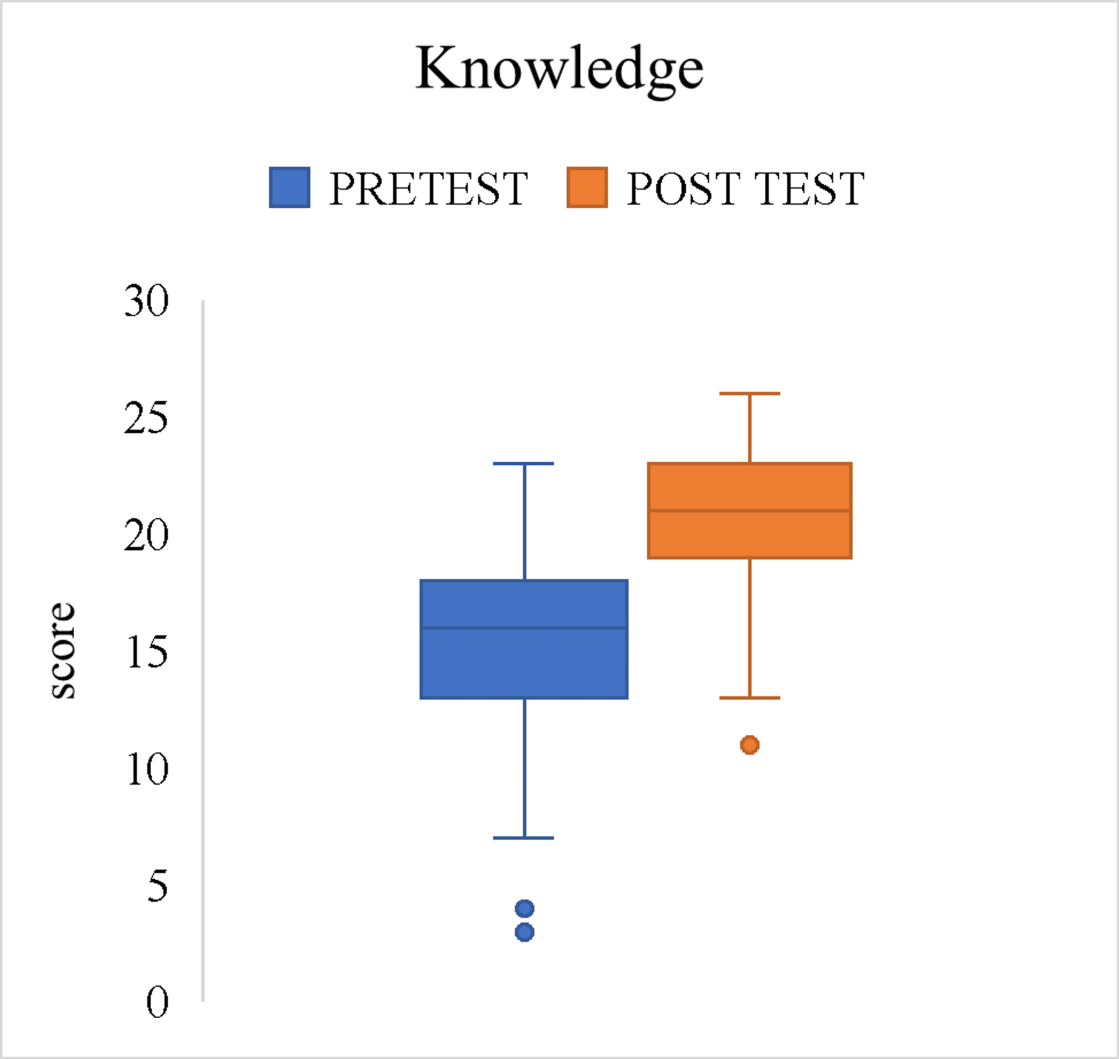
Effect of intervention on overall knowledge scores among the study participants (n = 500)

**Figure 2:**
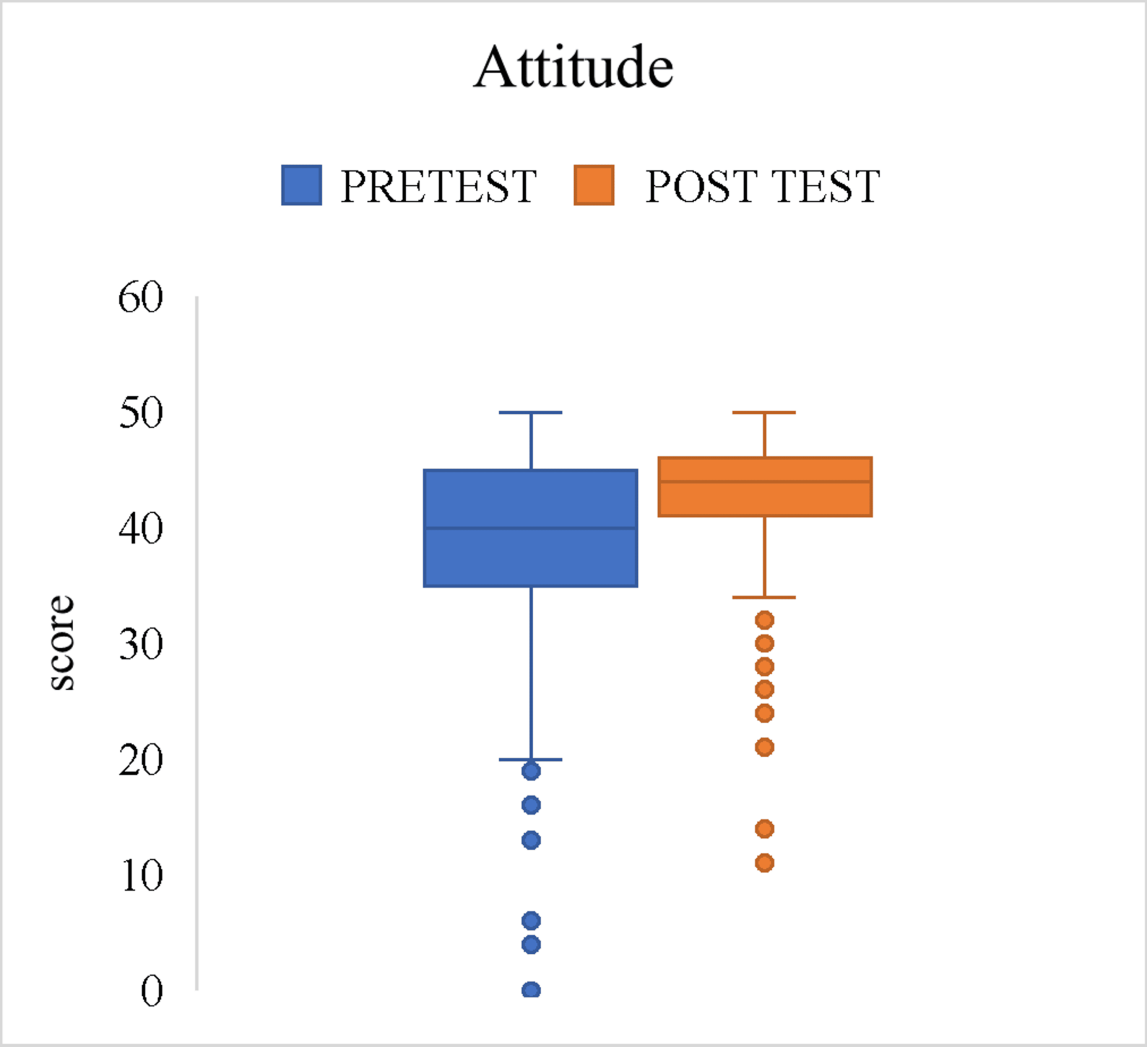
Effect of intervention on overall attitude scores among the study participants (n = 500)

The box and whisker plots display the distribution of knowledge and attitude scores, with separate plots for pre-test and post-test results. The effect size (r) for knowledge scores was 0.53, suggesting a moderate positive effect with a statistically significant difference (p < 0.001). For the attitude scores, the effect size (r) was 0.287, which indicates a small to moderate effect (p < 0.001), which is statistically significant (Figures 1-2).

## Discussion

The present study conducted among 500 PUC girls revealed that the mean age of the participants was 17.15 ± 0.7 years, and 98.6% were in the 16-18 years age group, which aligns with the study conducted by Pokhrel et al. in Belgaum, Karnataka [12] and Rao et al. in Udupi taluk, Karnataka [13], where 87% belonged to the same age group. In this study, the educational qualifications of the mothers of the participants revealed that 3.4% were illiterates, 17% completed eighth standard, 56% finished pre-university, and 23.6% were graduates/postgraduates. In contrast, a study done in Bhopal, Madhya Pradesh, found that the majority (53.29%) of the mothers of participants were illiterates, 30.45% had completed primary education, and only 16.24% had completed high school and college [11]. The difference in the results might be due to the variation in the literacy rate between each state. In the current study, 81.8% of participants had toilets inside their homes. Similarly, a study conducted in Bangladesh [14] showed only 51.7% of the participants reported having sufficient privacy in their toilets, and this difference might be due to the variation in the people’s living standards across different countries.

Regarding menstruation, in the present study, 70.4% of students got information from their mothers, 2.8% from the teachers, and 8.4% from their friends. This concurs with the study conducted by Nagaraj and Konapur, where mothers were the prime source of information for 47.03% of participants, followed by their sisters (25.65%) and friends (15.46%) [15]. Another similar study conducted in Turkey showed that 63% of girls obtained knowledge regarding menstruation from their mothers [16]. Most students felt comfortable in discussing menstruation-related issues with their mothers, and she can be a key person in imparting knowledge regarding the same.

In this study, during the pre-test, 49.6% of participants answered that menstruation is a physiological process, which increased to 81% in the post-test, and this aligns with a study done in Bengaluru where the knowledge regarding the same improved to 60.5% from 34.2% after intervention [15]. Initially, only 69.6% of participants knew the normal interval between two menstrual cycles (21-35 days), which improved to 85.2% after the health education intervention that mirrors the findings observed in the Bangladesh study [14], where knowledge regarding the duration of normal menstrual cycles (21-35 days) improved from 77.4% in the pre-test to 93.5% in the post-test, and another community-based study done in Tamil Nadu [17], where knowledge improved from 37.6% to 79%. 

In the present study, 79.8% of study participants stated that commercially made sanitary pads should be used during menstruation, which increased to 94% after the intervention, along with the frequency of changing absorbents (4-6 hours once), which was mentioned by 40.2% of participants in the pre-test, which increased to 60.6% in the post-test. The study conducted by Neelkanth et al. in Bhopal also highlighted that the usage of sanitary pads during menstruation increased from 58.3% to 69.5%, with the participants changing sanitary pads more than three times a day increasing from 40.10% to 94.41% [11]. In terms of disposal, during the pre-test, 89.20% of participants were aware that dustbins should be used, which improved to 95.4% in the post-test. Furthermore, 91.2% of participants were aware that they should clean the genitalia at every visit to the toilet during menstruation, which increased to 97.8% in the post-test. This lines up with the Coimbatore study where 30.5% of the girls had knowledge that they should wash their genitalia more often during menstruation, which improved to 66% in the post-test [18].

This study also revealed that only 39% of participants were aware that 18 years is the recommended marriage age for girls in India, which improved to 66.4% in the post-test. This finding equates to a study done by Ghongdemath et al. where 100% of participants correctly answered regarding the ideal marriage age in a post-test [19].

From this study, it was found that only 60.6% of the students had heard of contraception, which connects to a study conducted by Jain et al. in the Wardha district, Maharashtra [20], where only 49% of students knew about contraception. A similar study done in Vadodara, Gujarat [21] also reported that only 30% of students had knowledge about contraception. Initially, only 7.20% of students in this study were aware that contraception helps to maintain regular intervals between pregnancies, which increased to 45.60% in the post-test, and the results were almost on par with the study conducted by Kishor et al., where knowledge of the same increased from 29.3% to 85.3% after the health education intervention [10].

In the present study, during the pre-test, 33.6% of study participants strongly agreed that parents/teachers should be consulted regarding reproductive health, which improved to 54.8% in the post-test. About 41.4% of study participants felt cultural and social taboos during menstruation should be eliminated, which increased to 66% in the post-test. Cultural and social taboos can lead to misconceptions and hence should be avoided. Similarly, a study done by Phulambrikar et al. also showed an increase in positive attitudes from 34.3% to 54.3% [7]. The mean post-test knowledge score (20.97 ± 2.89) for reproductive health is significantly higher than the mean pre-test knowledge score (15.38 ± 3.827). This suggests that this health education program was successful. These findings align with a study by Parmar and Singh [22] and Maurya et al. [23], where the knowledge of menstrual hygiene was 13.01 ± 3.07 in the pre-test and 19.03 ± 1.57 in the post-test, statistically significant at p < 0.05. In a study conducted by Maharjan et al. [24], the post-test mean knowledge score of 15.8 ± 0.73 was substantially higher than the pre-test mean knowledge score of 13.36 ± 1.64, which again confirms the efficiency of the health intervention. The results of this research showed a noteworthy change in knowledge and attitude related to reproductive health when health education was implemented.

Limitations

This study was executed in an urban area; hence, it reflects the knowledge and attitude of PUC girls in urban areas. Additionally, the majority of the girls in high school were from upper and middle socioeconomic classes. The inclusion of participants from lower socioeconomic classes might possibly lead to more comprehensive conclusions. Here the participant group might reduce the generalizability of the outcomes to other populations.

## Conclusions

The present interventional study showed impressive improvement in knowledge and attitudes regarding reproductive health among the PUC girls in the urban area. The findings suggest that focused health education initiatives were successful in raising awareness about key aspects of reproductive health. Additionally, health education also has a good impact on attitudes, which results in more responsible conduct. The girls exposed to the educational interventions demonstrated a more open attitude toward discussing reproductive health issues, thereby decreasing the misconceptions, which highlights the importance of integrating comprehensive reproductive health education into school curricula, thus promoting better reproductive health outcomes. It was also found that the mothers of the participants play a pivotal role in imparting the right information on reproductive health. Therefore, more emphasis should be given to health education for parents, teachers, community, and peer education programs at a young age to improve the quality of reproductive health in adolescents.

## Appendices

Appendix A

Questionnaire for Pre-test and Post-test

“Effect of Health Education on Knowledge and Attitudes Toward Reproductive Health Among Pre-university Girls in an Urban Area”

SL.NO : _______      ROLL NO :____     DATE OF INTERVIEW :____________

 NAME OF THE PU COLLEGE:                                                                  

ADDRESS:

PART I - SOCIO DEMOGRAPHIC DETAILS:

1) Age            :    ____ years

2) Class         : 1. PUC 1    2. PUC 2

3) Religion   : 1. Hindu                 2. Muslim        3. Christian    4. others, specify_______

4) Educational qualification of Mother

     1.  Illiterate

    2.  Read and write

    3.  Standard 1-4

    4.  Standard 5-8

    5. 10-12 / Diploma

    6. Degree / Post graduate

5)  Occupation of the Mother

     1. Farmer

     2. laborer

     3. self employed

     4. Government  employee

     5. Private employee

     6. Retired / pensioner

     7. Home maker

6) Place of residence    :   1. Urban        2. Rural         3. Suburban             4. Hostel

7) Type of the family    : 1. Nuclear                     2. Joint                       

                                        3. Broken                       4. Three generation family.

8) Living with              : 1. Both parents     2. Mother alone      3. Father alone 

                                        4. Grand parents   5. If others, (specify) ________

9) What kind of house do you live? 

         1. Kutcha house (Made from grass or hay or mud)

         2. Semi-pucca (combination of mud, hay and bricks)

        3. Pucca (Built of cement/bricks with walls and roof)

10) What kind of toilet facility do you have?  

         1. We have a toilet inside our house

         2. We have a toilet built outside our house

         3. We use public toilet

         4. Don’t have a toilet. We practice open defecation.

11) Total monthly income                   : Rs __________

12)  Number of family members        : ___

13) Per capita income                         : Rs. ________/ month

14)  Socio economic status                 : (modified B.G. Prasad’s classification)

           1. Class I          2. Class II        3. Class III       4. Class IV      5. Class V

15) When did you attain your menarche?           ____ Years / Don’t Know

16) What were your physical symptoms when the first time you had menstruation?

       1. Abdominal and back pain   2. Sleeplessness 3. Heavy bleeding  4. Others, (specify)

17) From whom did you get information regarding menstruation? (multiple responses)

         1. Mother                             2. Teacher                                 3. Friends

        4. Books                              5. Media (TV, Radio)                6. Others, specify______

PART II - KNOWLEDGE:

A. PUBERTY AND MENSTRUATION:

1) What is menstruation?      1. Physiological          2. Pathological                3. Curse   

                                               4. Others,(Specify) ______________     5. Don’t know

2)  What do you think happens in menstruation or periods?

            1. Blood comes out through vagina among women

            2. Blood comes out through urinary opening among women

            3. Don’t know

3) Was there any session in your school where you were given information on menstruation?  YES   /  NO

4) Do you find comfortable talking about menstruation-related queries to your teacher?

        1. Yes, I can ask my queries to any teacher

        2. Yes but only to a teacher of same gender

        3. No, I feel uncomfortable to ask my queries to any teacher

5) What is the duration of normal menstruation?              ___Days  / Don’t know

6) What is the interval between two menstrual cycles?  (How long is it between one menstrual cycle to the  next?)___Days  / Don’t know

7)  How often should the pads be changed per day?

          1. 2-4 hours once         2. 4-6 hours once    3. 6-8 hours once         4. 8-12 hours once     5.12 hours once

8) what absorbent material should be  used during menstruation? (More than one response)

         1. Commercially made sanitary pad

         2. Napkin (soft paper)

         3. Cloth

        4.Others, (specify)________________

9)  Where do you think you should dispose your pads?

         1. Dustbin          2. Drain       3. Toilet         4. Open field       5. Other ______

10) What types of pad wraps should be used for disposing it?

        1. Papers          2. Plastic bag     3. Not wrap     4.others _____

11) Do you think you should clean your genitalia at every visit to toilet during menstruation? YES / NO   

B. PREGNANCY AND ANTENATAL CARE:

12) Recommended age of marriage for girls in India ______Years.  

13) First sign of pregnancy is missed period    YES / NO

14) Regular medical and nursing care is recommended for women during pregnancy            YES / NO 

15)  Proper diet, rest, and mild exercise are essential during pregnancy.            YES / NO

16) Eating nutritious foods is good for health of pregnant women and baby     YES / NO

C. CONTRACEPTION

17) Do you think it is necessary to know about contraception?    YES / NO / DON’T KNOW     

18) Who should be responsible for contraception?

    1. Husband                        3. Both of them have responsibility

    2.  Wife                             4.  Both of them don’t have responsibility

19) Contraception helps to avoid unwanted pregnancy                                YES / NO / DON’T KNOW

20) Contraception helps to maintain Regular interval between pregnancy     YES / NO / DON’T KNOW

PART III- ATTITUDE

**Table 7:** Attitude on puberty and menstruation

		Strongly disagree	Disagree	Neutral	Agree	Strongly agree
1	Parents/teachers should be consulted on the matters of reproductive health education.					
2	Do you agree that all girls need to learn about menstrual hygiene.					
3	Poor menstrual hygiene predisposes to infection.					
4	Does menstrual problems interfere with academic performance.					
5	Do you think that cultural and social taboos (not entering kitchen, do not sit in same place as others in house) during menstrual period should be eliminated.					

**Table 8:** Attitude on pregnancy and antenatal care

		Strongly disagree	Disagree	Neutral	Agree	Strongly agree
6.	Do you agree that marriage with in close relatives should be avoided.	
7.	Regular antenatal checkup and follow-up is essential during pregnancy.	
8.	Pregnant women should change dietary habit as advised by doctor.	
9.	Do you agree that taking medicine without prescription of doctors during pregnancy is harmful.	
10.	Smoking during pregnancy is harmful to baby.	
